# Development of the Middle Layer in the Anther of *Arabidopsis*

**DOI:** 10.3389/fpls.2021.634114

**Published:** 2021-02-10

**Authors:** Jing-Shi Xue, Chi Yao, Qin-Lin Xu, Chang-Xu Sui, Xin-Lei Jia, Wen-Jing Hu, Yong-Lin Lv, Yi-Feng Feng, Yu-Jia Peng, Shi-Yi Shen, Nai-Ying Yang, Yu-Xia Lou, Zhong-Nan Yang

**Affiliations:** Shanghai Key Laboratory of Plant Molecular Sciences, College of Life Sciences, Shanghai Normal University, Shanghai, China

**Keywords:** anther development, pollen, *Arabidopsis*, middle layer, living images

## Abstract

The middle layer is an essential cell layer of the anther wall located between the endothecium and tapetum in *Arabidopsis*. Based on sectioning, the middle layer was found to be degraded at stage 7, which led to the separation of the tapetum from the anther wall. Here, we established techniques for live imaging of the anther. We created a marker line with fluorescent proteins expressed in all anther layers to study anther development. Several staining methods were used in the intact anthers to study anther cell morphology. We clarified the initiation, development, and degradation of the middle layer in *Arabidopsis*. This layer is initiated from both the inner and outer secondary parietal cells at stage 4, stopped cell division at stage 6, and finally degraded at stage 11. The neighboring cell layers, the epidermis, and endothecium continued cell division until stage 10, which led to a thin middle layer. The degradation of the tapetum cell wall at stage 7 lead to its isolation from the anther wall. This work presents fundamental information on the development of the middle layer, which facilitates the further investigation of anther development and plant fertility. These live imaging methods could be useful in future studies.

## Introduction

The anther is the male reproductive organ in seed plants. Its main function is to produce and disperse pollen. Defects in anther development leading to male sterility are widely used in agriculture to increase crop yields (Tester and Langridge, 2010). In angiosperms, anther structure is generally conserved. The anther wall contains four layers called the epidermis, endothecium, middle layer, and tapetum. Inside anther locules, pollen mother cells undergo meiosis to form microspores enclosed in the tetrad. After release, microspores further develop into mature pollen (Goldberg et al., 1993; van der Linde and Walbot, 2019). Anther wall layers carry out different functions for pollen formation and release. The epidermis plays a protective role in anther development. The tapetum provides nutrients for microspore development and materials for pollen wall formation. The endothecium is responsible for anther dehiscence to disperse pollen when they are mature (van der Linde and Walbot, 2019). The middle layer is located between the tapetum and endothecium. Currently, knowledge of its development and function during anther development is limited.

*Arabidopsis* is the most celebrated model plant. Its anther development has been extensively investigated. The development of anthers in *Arabidopsis* is divided into 14 stages. The section technique is generally used to assess anther development. Based on this technique, the middle layer was shown to be a short-lived, transient cell type in *Arabidopsis* (Goldberg et al., 1993; Sanders et al., 1999; van der Linde and Walbot, 2019). This layer is established at stage 4 together with the four-lobed anther. The middle layer becomes thinner at stage 5 and is mechanically crushed or undergoes programmed cell death at stage 6 prior to the completion of meiosis. Only remnants of this layer are present at stage 7 when meiosis is finished and tetrads are formed. Finally, the middle layer completely disappears at stage 8 when the callose wall surrounding tetrads degenerates (Goldberg et al., 1993; Sanders et al., 1999; van der Linde and Walbot, 2019). However, by using transmission electron microscopy (TEM), the middle layer is observed at the late uninucleate microspore stage (Owen and Makaroff, 1995; Quilichini et al., 2014). Although current knowledge about the middle layer is quite limited, several mutants with defective middle layers have been reported. Loss-of-function mutants of several receptor-like kinases display defects in the middle layer and pollen formation (Mizuno et al., 2007; Cui et al., 2018). These mutant phenotypes indicate that the middle layer is essential for anther development and pollen formation.

The anther primordium cell layers are designated L1, L2, and L3. The endothelium, middle layer, and tapetum are derived from L2. L2 cells develop into archesporial cells, which divide into parietal cells and sporogenous cells. Parietal cells generate inner secondary parietal (ISP) cells and outer secondary parietal (OSP) cells (Goldberg et al., 1993; van der Linde and Walbot, 2019). OSP and ISP cells further develop into the endothecium and tapetum. The middle layer is located between the endothecium and tapetum. In a study of the *EXCESS MICROSPOROCYTES1* gene, the middle layer was observed to originate from OSP cells (Zhao et al., 2002; Zhao, 2009; Chang et al., 2011). In the study of tapetum development, ISP cells were shown to perform periclinal division to form the tapetum and the middle layer (Feng and Dickinson, 2010). Therefore, middle layer initiation still needs to be clarified.

Semithin sections observed by bright-field microscopy are the major method for cellular analysis of anther development. However, sections may damage the tissue morphology, and the resolution of bright-field microscopes is not sufficient to detect detailed information on anther development. Optical observation of intact plant tissue could be achieved by laser scanning confocal microscopy (LSCM). It provides high-resolution and dynamic images *in vivo* without fixing, sectioning, and dehydrating (Sappl and Heisler, 2013). However, plant tissues contain various components with different refractive indexes, leading to light scattering and low-quality LSCM images in the deep tissue (Kurihara et al., 2015). To enhance the signals in deep tissue, researchers have used transgenic marker lines expressing fluorescent proteins with LSCM. Several chemical reagents are used to “clear” (improve transparency and decrease light scattering) plant tissue. Through these treatments, cell patterning in the intact organ can be visualized (Kurihara et al., 2015). Many histological staining techniques are combined with one clearing method, ClearSee (Kurihara et al., 2015) and LSCM in intact tissues (Ursache et al., 2018). These methods improve the resolution of images and decrease the cost of fixing and sectioning. Together, these new methods have been used to visualize cell patterning in complex organs such as roots, leaves, or shoot apical meristems (SAMs) *in vivo* (Truernit et al., 2008; Andriankaja et al., 2012; Kurihara et al., 2015; Xue et al., 2015; Ursache et al., 2018).

The major method for anther cellular analysis is semithin sections of fixed samples (Cui et al., 2018; Jacobowitz et al., 2019; Xu et al., 2019; Yamamoto et al., 2020). In this study, we used an appropriate promoter for the ubiquitous expression of fluorescent proteins in anthers. A marker line for cell morphology was created for live imaging of the anther. Histological staining and clearing methods were also used with anthers to analyze the cell morphology. Through these methods, we clarified the initial process, development, and degradation of the middle layer in *Arabidopsis*. Further cell proliferation analyses showed the division patterns of different anther layers. Together, these studies provide fundamental knowledge of anther development. The application of new imaging techniques in anther development may provide further information for future studies.

## Results

### The Promoter of *POLYUBIQUITIN 10* Drives the Expression of Fluorescent Proteins in Cells of All Anther Layers

**FIGURE 1:**
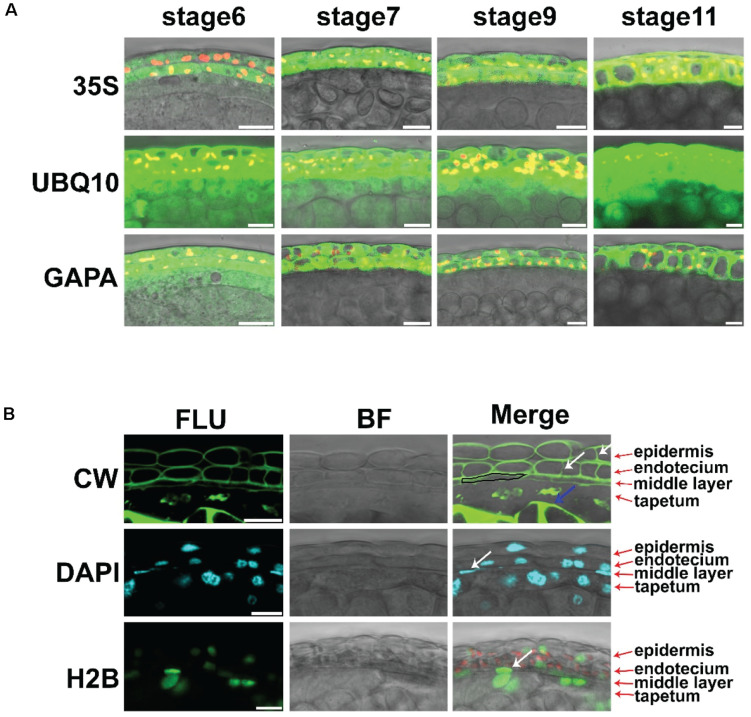
The live imaging methods for intact anther. **(A)** Expression of VENUS driven by the promoters of *35S, GAPA*, and *UBQ10* in anther stages 6, 7, 9, and 11. The VENUS signals are colored green, and the chloroplasts are colored red. Scale bar = 10 μm. **(B)** Fluorescence images of anthers stained with CW in anther stage 7 are shown in the first row. Arrows show the newly formed cell wall. The images of anthers stained with DAPI in anther stage 7 are shown in the second row. The images in the third row show the expression of *ProUBQ10-H2B-VENUS* in anther stage 7. The VENUS signals are colored green. The CW signals are colored green. The DAPI signals are colored cyan, and the chloroplasts are colored red; 35S: Pro35S: GFP. UBQ10: ProUBQ10: VENUS. GAPA: ProGAPA: VENUS. CW, Calcofluor white staining; DAPI, DAPI staining; H2B, ProUBQ10; H2B, VENUS; FLU, fluorescence signals; BF, bright field signals; Merge, merge signals. White arrows show the dividing cells and middle layer nucleus. The blue arrow shows the callose. The black box shows the middle layer cell. Scale bar = 10 μm.

The 35S promoter of the cauliflower mosaic virus is the most commonly used promoter in plants (Benfey and Chua, 1990). We introduced the *Pro35S:GFP* transgenic construct into *Arabidopsis*. GFP signals could be detected in the leaf epidermis, mesophyll, anther epidermis, and endothecium (Supplementary Figure 1 and Figure 1A). However, no GFP signal was detected in the middle layer, tapetum, or microspores of these transgenic lines (Figure 1A). To screen appropriate promoters for the *in vivo* analysis of anthers, we analyzed the expression of several housekeeping genes in anthers (Supplementary Figure 2). Among these genes, *TUBULINA6* (*TUA6*), *TUBULIN6* (*TUB6*), *GLYCERALDEHYDE 3 PHOSPHATE DEHYDROGENASE A SUBUNIT* (*GAPA*), and *POLYUBIQUITIN 10* (*UBQ10*) were highly expressed in anthers. We cloned the promoters of these genes to generate the constructs *ProTUA6: VENUS*, *Pro TUB6: VENUS*, *ProGAPA: VENUS*, and *ProUBQ10: VENUS*. After introduction into wild-type *Arabidopsis*, at least three independent transgenic lines for each construct were analyzed. Through LSCM, VENUS signals were detected in the leaf epidermis and mesophyll of all these transgenic lines (Supplementary Figures 1, 3). However, no VENUS signals of TUA6-VENUS, TUB6-VENUS, or GAPA-VENUS were detected in the tapetum, microspore mother cells, or microspores of these plants (Figure 1A and Supplementary Figures 1, 3). Thus, the *TUA6*, *TUB6*, and *GAPA* promoters could not drive VENUS expression in every anther layer. In the *ProUBQ10:VENUS* transgenic plants, VENUS signals were detected ubiquitously at stages 4–5 in anthers (Supplementary Figure 1). At stages 6–10, strong VENUS signals were detected in the epidermis, endothecium, and middle layer (Figure 1A). Thus, the *UBQ10* promoter conferred ubiquitous expression in anthers. This promoter could be used to construct marker lines for anther developmental analysis.

### The Establishment of Live Imaging Methods in Intact Anthers

Plant tissues with different compositions can cause light scattering. This phenomenon leads to difficulty in observing deep plant tissue using LSCM. In this study, the clearing method of ClearSee combined with the cell wall polysaccharide dye Calcofluor white (CW) (Ursache et al., 2018), as described in another study. The CW signals clearly showed the cell wall of the anther layers at stage 7 (Figure 1B). Newly formed cell walls showed weak signals in the epidermis and endothecium layer (Figure 1B). The middle layer was thinner than its neighboring layers (Figure 1B). The tapetum cell wall was degraded (Figure 1B). Strong CW signals of the callose surrounding tetrads were identified in the anther locules (Figure 1B). Together, the different cell types of anthers were clearly identified. The classic dye 4′6-diamidino-2-phenylindole (DAPI) is used to stain DNA in nuclei. Here, a simplified clearing method (see “Materials and Methods” section) was combined with DAPI staining to show the nuclei in intact anthers. Through this staining, the epidermis, endothecium, tapetum, and tetrads showed typical rounded nuclei, while oblong nuclei were observed in the middle layer (Figure 1B). Only the tapetum contains two nuclei at this stage (Figure 1B). Histone 2B (H2B) is a marker protein of nucleosomes. To confirm the DAPI staining, we constructed *ProUBQ10*:*H2B*:*VENUS* and transferred it into wild-type *Arabidopsis* to visualize the nucleosomes. The VENUS signals were detected by LSCM and showed a similar shape to DAPI in the transgenic plants *in vivo* (Figure 1B). Together, these methods clearly showed the characteristics of the cell layers in the intact anther of *Arabidopsis*.

### The Middle Layer Is Derived From Both Inner Secondary Parietal (ISP) and Outer Secondary Parietal (OSP) Cells in the Anther

**FIGURE 2:**
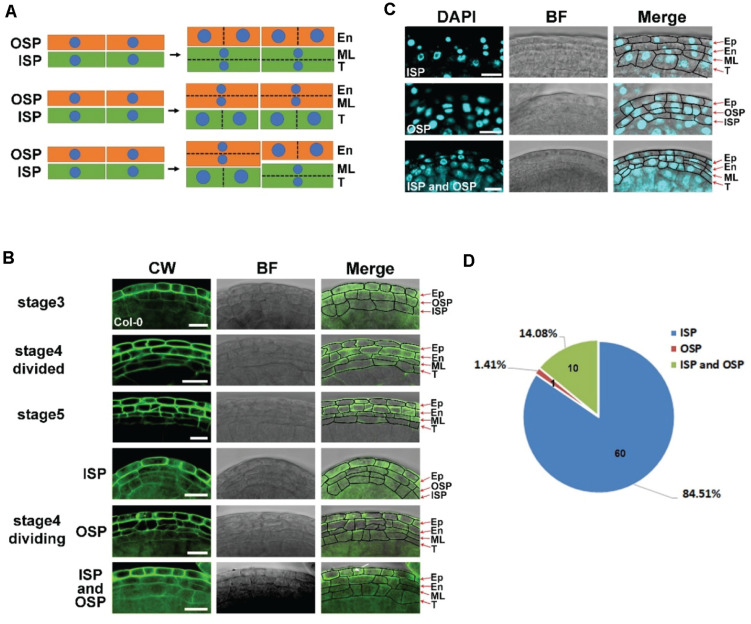
The middle layer is derived from both inner secondary parietal (ISP) and outer secondary parietal (OSP) cells in the anther. **(A)** Model of the origin of the middle layer. The first model shows periclinal division in ISP cells to form the endothecium and middle layer. The second model shows periclinal division in OSP cells to form the tapetum and middle layer. The last model shows periclinal division in both OSP and ISP cells. **(B)** Images of Col-0 anthers stained with CW at stages 3, 4, and 5. The images in the left column show the CW signals. The images in the middle showed bright field signals. The right images are the merged signals of Calcoflour white (CW) and the bright field. Arrow shows the newly formed cell wall. Stage 4 divided: the anther finished periclinal division at stage 4 with the middle layer fully formed. Stage 4 division: the anther was undergoing periclinal division. The middle layers were derived from inner secondary parietal cells (ISPs), outer secondary parietal cells (OSPs), and both inner secondary parietal cells and outer secondary parietal cells (ISPs and OSPs) in the same anther. Scale bar = 10 μm. **(C)** Fluorescence images of Col-0 anthers stained with 4′,6-diamidine-2-phenylindole (DAPI) at stage 4. The images in the left column show the DAPI signals. The images in the middle showed bright field signals. The right images merge the DAPI signals and the bright field signals. Scale bar = 10 μm. **(D)** The statistics of CW staining anthers undergoing periclinal division in stage 4. ISP: middle layer derived from inner secondary parietal cells. OSP: middle layer derived from outer secondary parietal cells. ISP and OSP: middle layer derived from both inner secondary parietal cells and outer secondary parietal cells in the same anther.

ISP and OSP cells are derived from parietal cells at stage 3 (Sanders et al., 1999). Generally, anticlinal division increases the cell number of each layer, while periclinal division leads to the formation of new cell layers. Direct observation of the axial cell division could be based on nuclear division directions and the axial direction of the newly formed cell wall. The middle layer is established at stage 4 (Sanders et al., 1999). After the establishment of this layer, the original axial cell division could not be detected. To identify anthers that were at stage 4 and were undergoing periclinal division, we analyzed the relationship between flower size and anther stages (Supplementary Figure 4). Anthers of approximately 339 μm^2^ that were at stage 4 were collected. LSCM was used to identify the maximum longitudinal section of each anther (Supplementary Figure 5). In this section, the axial division of the ISP and OSP layers was analyzed in *Arabidopsis* ecotype Col-0 (Figure 2A). CW staining was used to show the axis of the newly formed cell wall. We identified 71 anthers that had established their middle layer (Figure 2D). The signals of newly formed cell walls that separated two daughter cells were detected in all the cell layers (Figure 2B). In the epidermis, newly formed cell walls were perpendicular to the long side (Figure 2B), indicating anticlinal division in these layers. However, in ISP and OSP cells, newly formed cell walls were perpendicular to both the short side and long side in different cells (Figure 2B). These results showed that periclinal division appeared in both ISP and OSP cells, indicating that the middle layer was derived from both layers. To further confirm this result, we performed DAPI staining. Consistent with the CW staining, DAPI signals increased by periclinal division were also detected in both the ISP and OSP layers (Figure 2C). Thus, evidence of nuclear division and newly formed cell walls consistently showed that the middle layer originates from both ISP and OSP cells. Statistics showed that the middle layer was generated from ISP cells in 84.51% of the anthers, from both ISP and OSP cells in 14.08% of the anthers, and from OSP cells in 1.41% of the anthers in Col-0 (Figure 2D). This result indicates that the initial middle layer varies in Col-0.

### The Cell Division and Expansion of the Middle Layer

**FIGURE 3:**
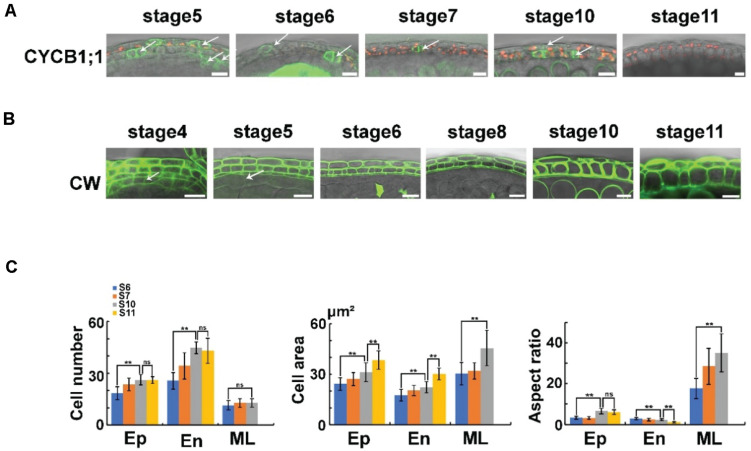
The cell division and expansion of anther layers. **(A)** The expression of *ProCYCB1;1*: *CYCB1;1*: *VENUS* in anther stages 5–11. The VENUS signals are colored green, and the chloroplasts are colored red. Arrows show the dividing cells. Scale bar = 10 μm. **(B)** The CW staining in anther stages 4–11. The CW signals are colored green. Arrows show the tapetum cell wall. Scale bar = 10 μm. **(C)** Statistical analysis of anthers in anther stages 6, 7, 10, and 11. The first image on the left shows the cell number. The second image shows the cell area. The third shows the aspect ratio. For each layer, the cell number was quantified from at least 10 anthers per stage with three biological repeats. The cell area and aspect ratio were quantified from five cells in each layer. At least 10 anthers per stage were used for the analysis with three biological repeats. Further statistical analysis was performed by R. Asterisks indicate statistically significant differences (*P* < 0.01, ANOVA by R). Ep, epidermis; En, endothecium; ML, middle layer; S6, stage 6; S7, stage 7; S10, stage 10; S11, stage 11; Ns, not significant.

Sections reveal that the morphology of the middle layer is similar to that of the tapetum at stage 4, while this layer becomes thinner at stages 5–6 and finally cannot be detected at stage 8 (Sanders et al., 1999). Cell division and expansion are critical for cell morphology and organ development (Anastasiou and Lenhard, 2007). To study cell division in anthers, we introduced a cell cycle marker into *Arabidopsis*. B-type CYCLIN (CYCB) is a key factor in the G_2_/M transitions (De Veylder et al., 2007). *ProCYCB1;1*: *CYCB1;1*: *VENUS* was constructed and transferred to wild-type Col-0 to generate a proper marker line. VENUS signals were detected at all the cell layers before stage 6 in these transgenic plants (Figure 3A). At stage 6, the VENUS signals of CYCB disappeared in the middle layer and tapetum. However, in the epidermis and endothecium, the signals of the marker protein were detected until stage 10 (Figure 3A). These results indicated that the cell division of the middle layer and tapetum stops at stage 6, while the epidermis and endothecium undergo cell division from stage 5 to 10. To confirm these results, we determined the cell number in the anther layer. CW staining was performed in intact anthers at stages 6–10. At the maximum longitudinal sections (Supplementary Figure 5), the cell numbers of each layer were counted (Figure 3B). In the middle layer, no significant cell number increase was detected from stage 6 to 10 (Figure 3C). However, the endothecium and epidermis cell numbers showed a significant increase in these stages (Figure 3C). Taken together, these results showed a distinct cell division regulation of anther layers.

Anther size dramatically increased in stages 4–11 (Supplementary Figure 4). To study the correlated cell expansion, we analyzed cell areas of the epidermis, endothecium, and middle layer. The cell area of the middle layer showed no increase at stages 6–7 (Figure 3C). In stages 7–10, the cell area of middle layer cells was increased significantly (Figure 3C), suggesting that cell expansion occurred in these stages. The cell areas of the endothecium and epidermis showed a significant increase from stage 5 to 10 (Figure 3C), together with the results of the cell cycle marker, indicating that both cell division and cell expansion occurred in the endothecium and epidermis at these stages. Taken together, after the establishment of anther layers at stage 4, the middle layer underwent cell division at stages 4–6 and cell expansion at stages 7–10. The cells of the endothecium and epidermis layer underwent both cell division and cell expansion at stages 5–10 (Figure 3). The side length of the anther cells was measured to analyze the cell shape of each layer. The aspect ratio of the middle layer was dramatically increased (Figure 3C). This increase leads to an increasingly thin middle layer that is hard to detect in sections after stage 8 (Sanders et al., 1999). The epidermal and endothelial cells showed a similar aspect ratio at stage 6 (Figures 3B,C). However, in stages 7–11, the aspect ratio was increased in the epidermis but decreased in the endothecium (Figure 3C), resulting in a thin epidermis and a thick endothecium layer. These results showed a distinct regulation of cell expansion in different anther layers.

### The Degradation of the Middle Layer Started at Stage 10

**FIGURE 4:**
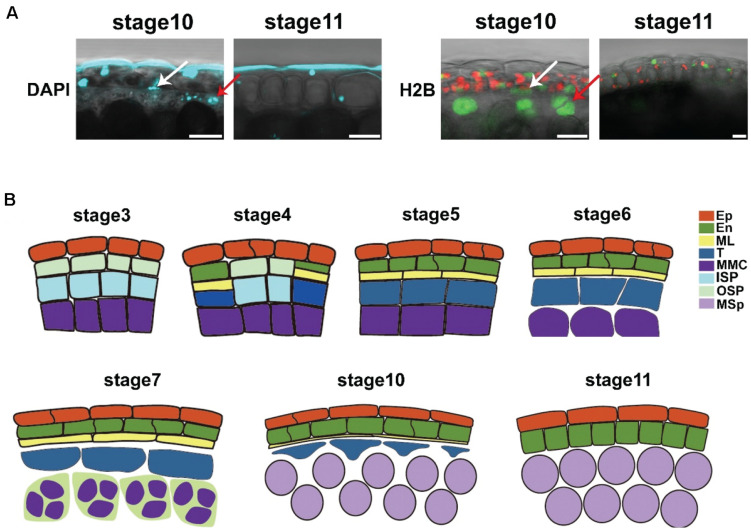
The degradation of the middle layer started at stage 10. **(A)** DAPI staining and ProUBQ10-H2B-VENUS signals in anther stages 10 and 11. Arrows show the middle layer and tapetum cells. The VENUS signals are colored green, the chloroplasts are colored red, and the DAPI signals are colored cyan. DAPI, DAPI staining; H2B, ProUBQ10-H2B-VENUS. Scale bar = 10 μm. **(B)** Model of anther development. Images showed anther cell morphology during anther development. Ep, epidermis; En, endothecium; ML, middle layer; T, tapetum; MMC, microspore mother cell; ISP, inner secondary parietal cell; OSP; outer secondary parietal cell; MSp, microspores.

The middle layer is considered to be degraded at stage 7 (Goldberg et al., 1993; Sanders et al., 1999; Walbot and Egger, 2016). However, in the intact anther, our results showed that the middle layer was not degraded in this stage (Figure 3B), which was consistent with previous reports (Owen and Makaroff, 1995; Quilichini et al., 2014). The cell size of the middle layer was still increasing during stages 7–10 (Figures 3B,C). To study the degradation of the middle layer, we performed CW and DAPI staining in *Arabidopsis* to individually show the degradation of the cell wall and nucleus. DAPI staining showed that the DNA signals in the nucleus were still detectable at stage 10 (Figure 4A). In stage 11, no DAPI signals could be detected in the middle layer (Figure 4A). Consistently, CW staining showed that signals in the cell wall of the middle layer could be detected at stage 10 but completely disappeared at stage 11 (Figure 3B). To further confirm the staining results, we used the marker line *ProUBQ10*: *H2B*: *VENUS*. The H2B-VENUS signals showed similar results with DAPI staining (Figure 4A). Together, evidence from both cell wall and nuclear degradation studies demonstrated that the middle layer survived until stage 10 and completely disappeared at stage 11. This finding is similar to the degradation of the tapetum. Thus, we showed that the middle layer and tapetum were degraded at the same stage.

## Discussion

Anther development is critical for pollen formation. The traditional evidence of anther development is mainly from semithin sections (Zhao et al., 2002; Feng and Dickinson, 2010). In this work, we developed several live imaging methods for the study of anther development. After screening the ubiquitously expressed promoter UBQ10, we created the marker line *ProUBQ10*: *H2B*: *VENUS*, which is a useful tool in the study of cell morphology and proliferation. The marker line is not only used for live imaging analysis of the middle layer in this work but could also be used in the study of other layers in anthers. In this study, we used CW and DAPI staining in intact anthers to avoid damage to sections. These easy-to-use and low-cost methods provide better images than current methods for developmental studies *in vivo*. Through these methods, we clearly showed the developmental process of the middle layer. Other anther layers could also be analyzed through these methods.

L2 anther primordium cells develop into parietal cells, which further generate ISP and OSP cells (Goldberg et al., 1993; Sanders et al., 1999). Middle layer formation is studied in both monocotyledon plant maize and rice, and dicotyledon plant *Arabidopsis*. In maize, OSP cells are considered to only undergo anticlinal divisions to form the endothecium layer, and ISP cells undergo periclinal divisions to produce both the middle layer and tapetum (Sheridan et al., 1999). In rice, the middle layer is also considered to be divided only from ISP cells (Raghavan, 1988). Thus, the identity of individual cell layers is proposed to be determined by genetically controlled periclinal cell divisions (Kelliher and Walbot, 2011). However, in *Arabidopsis*, OSP cells are also reported to generate middle layer cells (Zhao et al., 2002; Feng and Dickinson, 2010). In this study, we used staining methods to analyze the initiation of the middle layer in *Arabidopsis*. Evidence of both nuclear division and new cell wall formation showed that the middle layer was derived from both the ISP and OSP cells in Col-0 (Figure 2), indicating that the cell fate determination of the middle layer was non-cell-autonomous in *Arabidopsis*.

Cell division and expansion are the basis of anther size enlargement. In this study, we showed that different anther layers exhibited distinct cell proliferation and expansion strategies to support anther development. For the epidermis and endothecium, cell division is maintained for a long time, providing enough cells to build anther locules with enough space for microspore development and pollen formation. The endothecium is required for the anther dehiscence. This layer is lignified at stage 11 to provide resistance to bending and stretching, and is essential for final anther dehiscence (Steiner-Lange et al., 2003; Yang et al., 2007, 2017; Xu et al., 2019). For this layer, more cell divisions occur than in the epidermis. The aspect ratio of this layer decreases from stage 5 to 11, forming a quadratic cell layer. These quadratic types of cells provide a thick layer, which may provide more strength for anther dehiscence after lignification of their cell walls.

The middle layer is very thin at stages 6–7 and is considered to be degraded before the completion of meiosis, as shown by semithin sections (Goldberg et al., 1993; Sanders et al., 1999; van der Linde and Walbot, 2019). Transverse sections showed that the tapetum is usually isolated from the anther wall at stage 8 (Sanders et al., 1999). The degradation of the middle layer is believed to separate the endothecium from the tapetum (van der Linde and Walbot, 2019). We showed that the middle layer was undergoing cell division with an increasing aspect ratio at stages 4–6, which led to a very thin middle layer that was hard to detect in the semithin sections (Figure 3). However, this layer survived after meiosis, and the cell size was still increasing with an even higher aspect ratio at stages 7–10. These results showed that this layer existed much longer than previously believed. The tapetum provides material and nutrition for microspore development and pollen formation (Zhu et al., 2011). Through the sections, the tapetum was found to be isolated from the anther wall and detected in the anther locules at stages 7–10 (Sanders et al., 1999). The degradation of the middle layer is assumed to be responsible for the dissociation of the tapetum from the anther wall and avoids the direct influence of the endothecium on the tapetum (van der Linde and Walbot, 2019). However, we showed that the middle layer was not degraded until stage 11. By staining semithin sections, the cellulosic wall of the tapetum is degraded before the onset of meiosis (Matsuo et al., 2013). We observed the cell wall of the tapetum through CW staining in intact anthers. The signals of the tapetum cell wall were normally detected at stage 4 (Figure 3B). However, CW signals of the tapetum cell wall dramatically decreased at stage 5 (Figure 3B) and disappeared at stages 67 (Figures 1B,3B). This result was consistent with a previous investigation (Matsuo et al., 2013) that showed that the tapetum cell wall was degraded at stage 7. The degradation of the tapetum cell wall may lead to the isolation of this layer from the anther wall. Taken together, these studies provide fundamental information on anther development (Figure 4B), which is important for pollen formation and plant reproduction.

## Materials and Methods

### Plant Materials and Growth Conditions

Plants were grown under long-day conditions (16 h light/8 h dark) in an ∼22°C growth room. Ecotype Col-0 was obtained from ABRC.

### Plasmid Construction and Plant Transformation

For generation of *Pro35S-GFP*, *ProTUA6-VENUS*, *ProTUB6-VENUS*, *ProGAPA-VENUS*, and *ProUBQ10-VENUS*, the promoters of *35S, TUA6, TUB6, GAPA*, and *UBQ10* were fused with the P1300-VENUS plasmid by using the pEASY Uni-Seamless Cloning and Assembly Kit (Yao et al., 2018). For generation of *ProUBQ10-H2B-VENUS*, the genomic DNA of *H2B* was fused with the P1300-UBQ10-VENUS plasmid by using the same method as above. For generation of *ProCYCB1;1-CYCB1;1-VENUS*, the genome of CYCB1;1 was fused with the P1300-VENUS plasmid. For plant transformation, the plasmid was introduced into *Agrobacterium* strain GV3101 and transformed into plants by the floral dip method (Clough and Bent, 2010).

### DAPI Staining

DAPI (4′,6-diamidino-2-phenylindole) solution was prepared as previously described (Scali and Tinti, 1992). Briefly, flowers without petals and calyxes were dissected and immersed in DAPI solution, vacuumed for 20 min and stained at 4°C in the dark for 48 h. After this treatment, the transparency was increased, and DAPI signals in intact anthers were visualizable.

### CW Staining

ClearSee, a clearing agent, was prepared as previously described (Ursache et al., 2018). Briefly, flowers without petals and calyxes were dissected and treated with ClearSee solution for 7 days. The treated anthers were then stained with 2 mg/ml Calcofluor white (Fluorescent brightener 28) (Sigma, cat# 4404-43-7) in ClearSee for 6 h (Ursache et al., 2018). The anther was washed five times using ClearSee solution before analysis.

### Confocal Microscopy

All fluorescence images were obtained with an Olympus FV3000 laser scanning microscope. For monitoring of VENUS signals, a 514 nm laser line was used for excitation, and a 525–570 nm bandpass filter was used for detection (Xue et al., 2020). For GFP fluorescence, a 488 nm laser was used for excitation, and a 498–532 nm bandpass filter was used for detection. For DAPI signals, we used 405 nm for excitation, and emission spectra were recorded in the range of 425–475 nm. For the CW signals, we used 405 nm for excitation, and emission spectra were recorded at 430–470 nm. For the cell division, cell number, and cell area analyses, we used z-stacks to identify the maximum light cross-sections of every intact anther. Then, ImageJ was used to analyze the cell area, aspect ratio, and cell number.

## Data Availability Statement

The original contributions presented in the study are included in the article/Supplementary Material, further inquiries can be directed to the corresponding author/s.

## Author Contributions

Z-NY and J-SX conceived and designed the experiments. CY, J-SX, X-LJ, S-YS, C-XS, Y-JP, Q-LX, Y-FF, N-YY, Y-LL, and W-JH performed the experiments. J-SX, CY, and Z-NY analyzed the data. J-SX, CY, Y-XL, and Z-NY wrote the manuscript. All authors have read the manuscript and approved it for submission.

## Conflict of Interest

The authors declare that the research was conducted in the absence of any commercial or financial relationships that could be construed as a potential conflict of interest.

## Supplementary Material

Supplementary Figure 1 Expression of the promoters of *35S, GAPA*, and *UBQ10* in leaf epidermis, mesophyll, and anther stage 4. The VENUS signals are colored green, and the chloroplasts are colored red. Ep: epidermis. Me: mesophyll. 35S: Pro35S: GFP. UBQ10: ProUBQ10: VENUS. GAPA: ProGAPA: VENUS. Scale bar = 10 μm.

Supplementary Figure 2 Expression levels of several housekeeping genes in the anthers of young flowers. The expression data is obtained from TraVA (travadb.org).

Supplementary Figure 3 Expression of Pro*TUA6* and Pro*TUB6* in leaf and anther. Expression of the promoters of *TUA6* and *TUB6* in leaf epidermis, mesophyll, and anther stages 4, 6, 7, 9, and 11. The VENUS signals are colored green, and the chloroplasts are colored red. Ep: epidermis. Me: mesophyll. TUA6: ProTUA6: VENUS. TUB6: ProTUB6: VENUS. Scale bar = 10 μm.

Supplementary Figure 4 The relationship between flower size and anther stages during anther development. **(A)** The top row shows the images of buds from stage 4 to stage 11, and the bottom row shows the images of anthers from stage 4 to stage 11. Scale bars = 1 mm in the first row and 20 μm in the bottom row. **(B)** Statistical analysis of the anther area.

Supplementary Figure 5 The identification of the maximum light cross-section of intact anthers in stage 7. **(A)** The LSCM anther analysis model for cell division, cell area, and cell morphology. A Z-stack was performed to identify the maximum light sections of the intact anther. **(B)** The z-stack of a stage 7 anther. The epidermis layer was artificially set as 0 μm. The step size for the z-stack was 8 μm. Arrows show the region with four anther wall layers in the maximum light sections of the intact anther. Cell number counting was performed in this region. Five cells in the middle of the anther wall were used for the cell area and aspect ratio analysis. The CW signals were colored green. Scale bar = 10 μm. **(C)** Statistical analysis of the anther locule area in light sections for the identification of the maximum light section. The maximum light section was further used for other analyses.
