# Supplementary figures and images for: Development of the Middle Layer in the Anther of Arabidopsis
Source: Front Plant Sci. 2021 Feb 10;12:634114. doi: 10.3389/fpls.2021.634114 (PMC7902515; doi:10.3389/fpls.2021.634114)

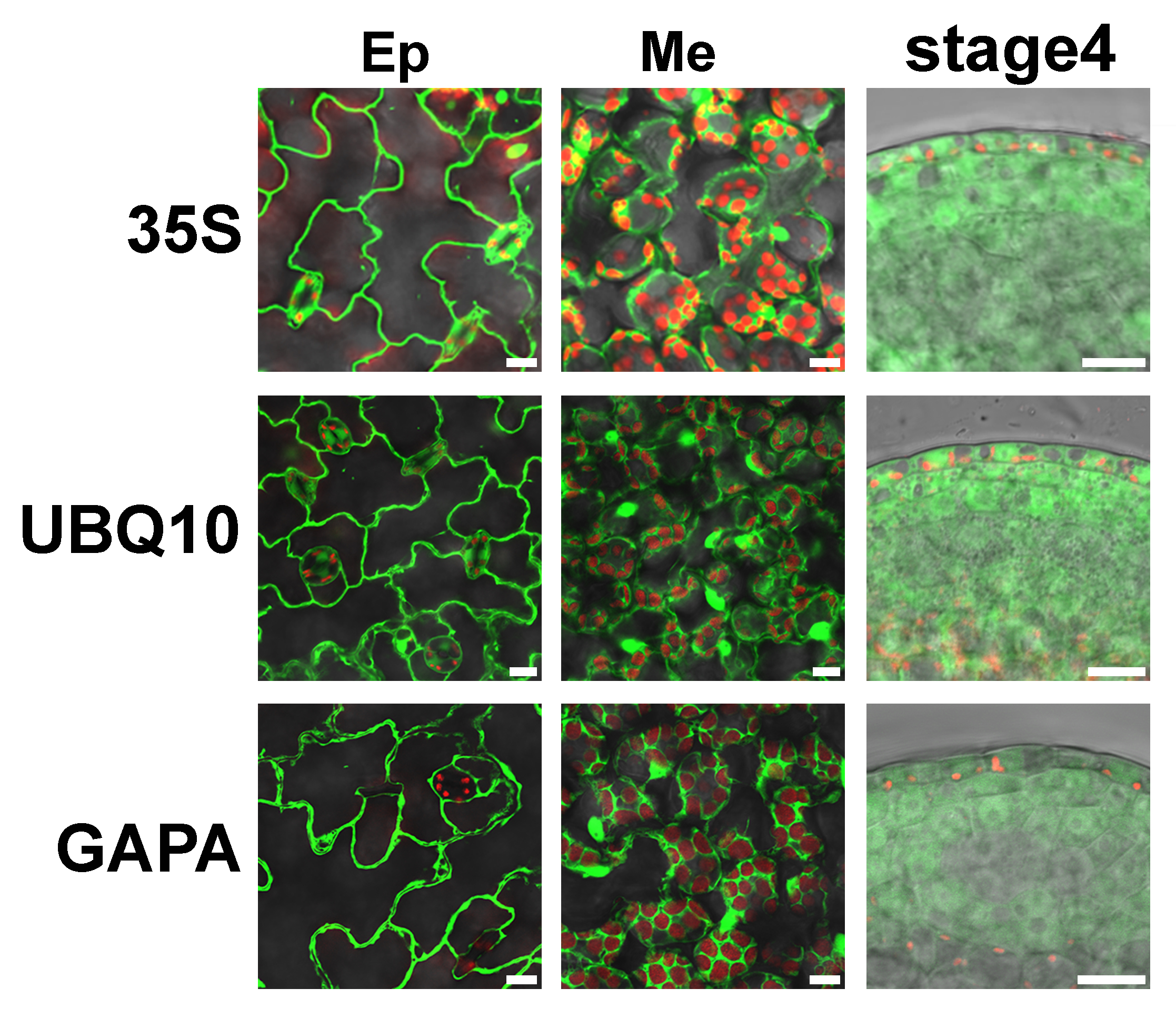

Supplement: Supplementary Figure 1 — Expression of the promoters of 35S, GAPA, and UBQ10 in leaf epidermis, mesophyll, and anther stage 4. The VENUS signals are colored green, and the chloroplasts are colored red. Ep: epidermis. Me: mesophyll. 35S: Pro35S: GFP. UBQ10: ProUBQ10: VENUS. GAPA: ProGAPA: VENUS. Scale bar = 10 μm. [file Image_1.png]

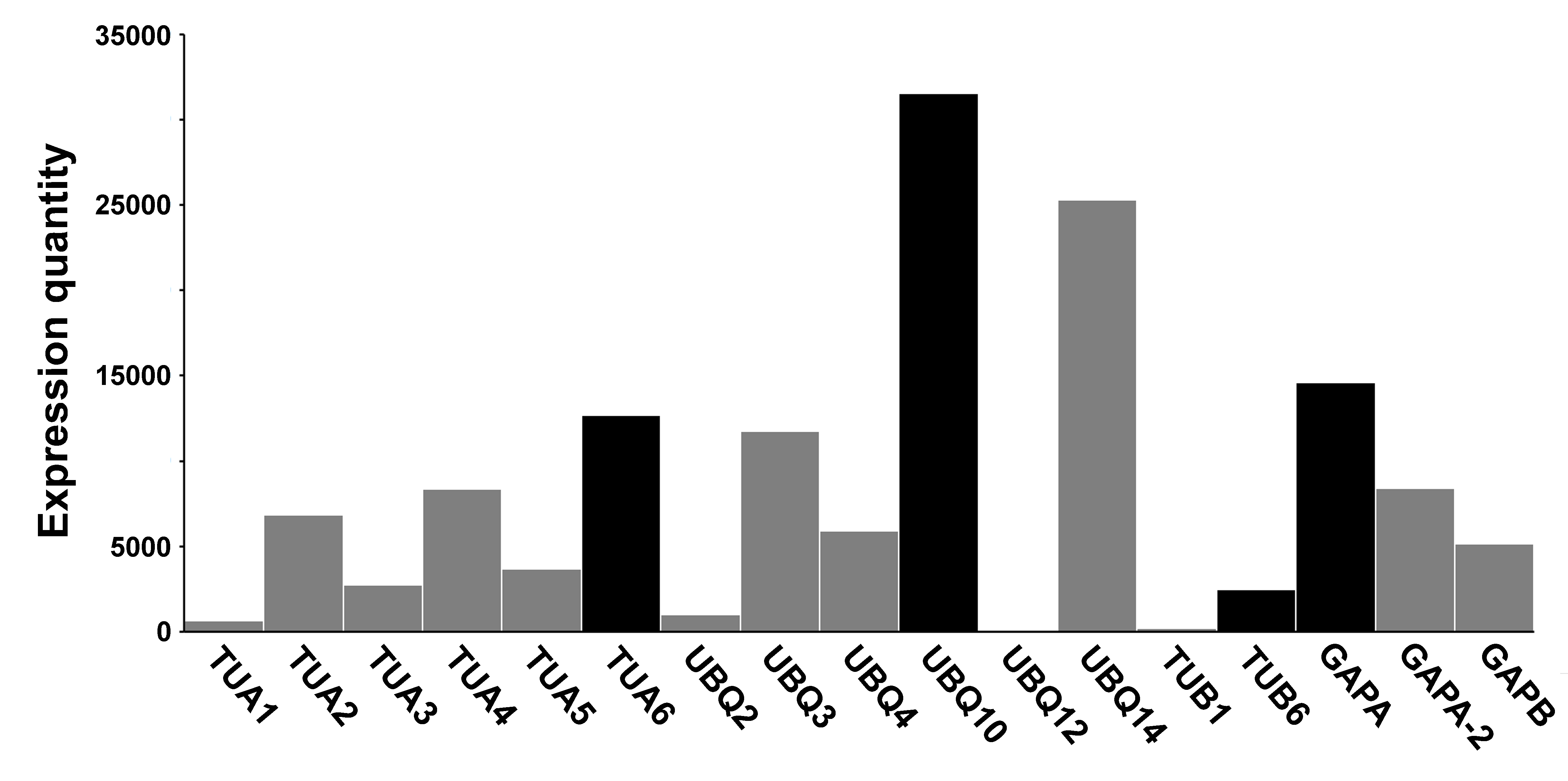

Supplement: Supplementary Figure 2 — Expression levels of several housekeeping genes in the anthers of young flowers. The expression data is obtained from TraVA (travadb.org). [file Image_2.png]

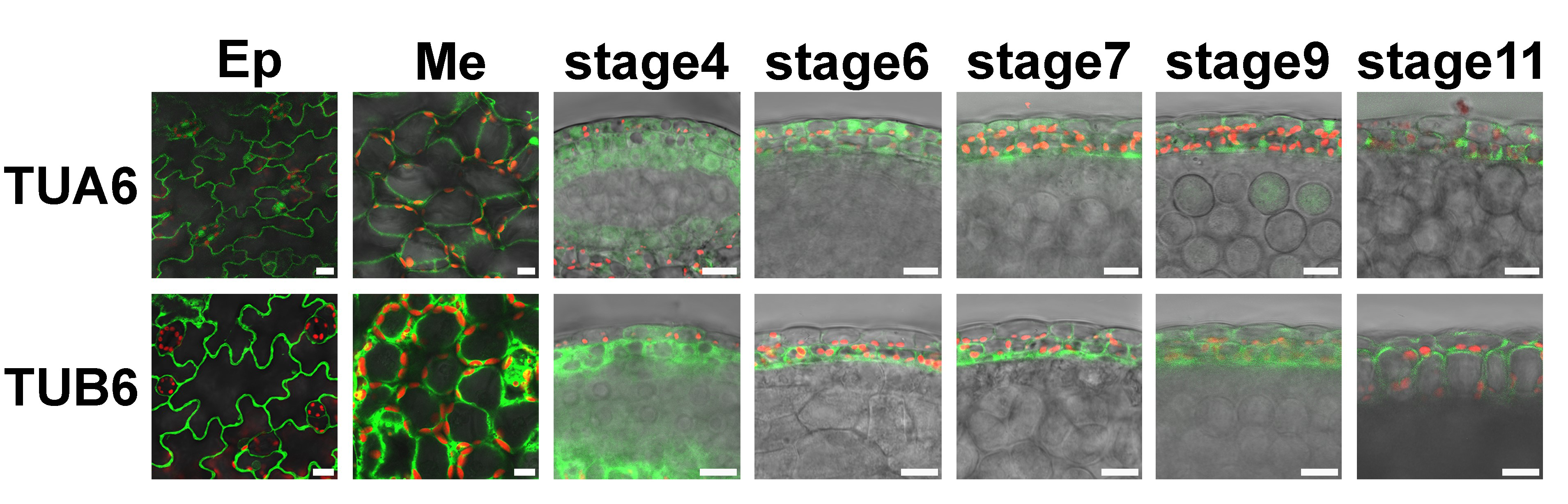

Supplement: Supplementary Figure 3 — Expression of ProTUA6 and ProTUB6 in leaf and anther. Expression of the promoters of TUA6 and TUB6 in leaf epidermis, mesophyll, and anther stages 4, 6, 7, 9, and 11. The VENUS signals are colored green, and the chloroplasts are colored red. Ep: epidermis. Me: mesophyll. TUA6: ProTUA6: VENUS. TUB6: ProTUB6: VENUS. Scale bar = 10 μm. [file Image_3.png]

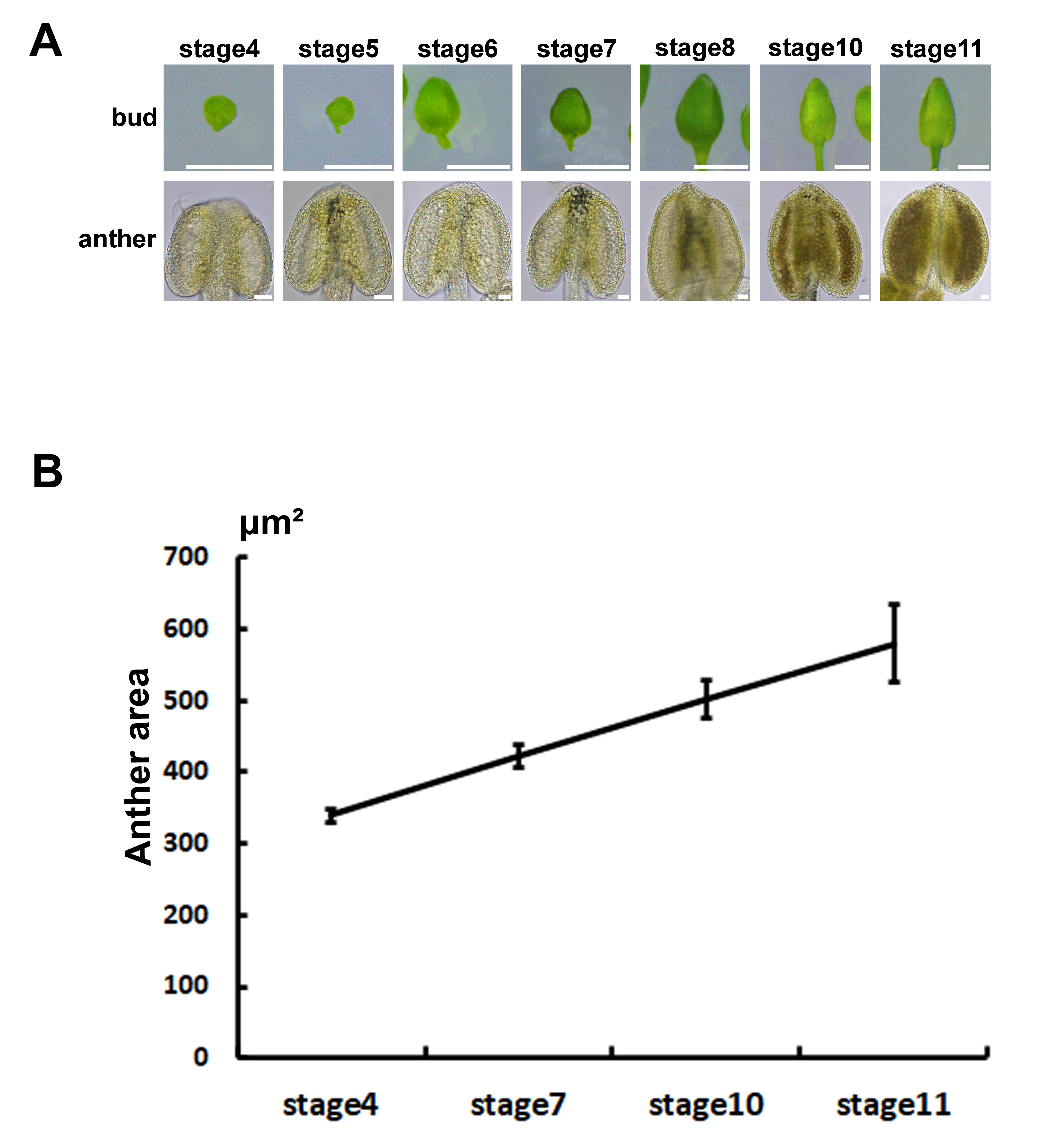

Supplement: Supplementary Figure 4 — The relationship between flower size and anther stages during anther development. (A) The top row shows the images of buds from stage 4 to stage 11, and the bottom row shows the images of anthers from stage 4 to stage 11. Scale bars = 1 mm in the first row and 20 μm in the bottom row. (B) Statistical analysis of the anther area. [file Image_4.png]

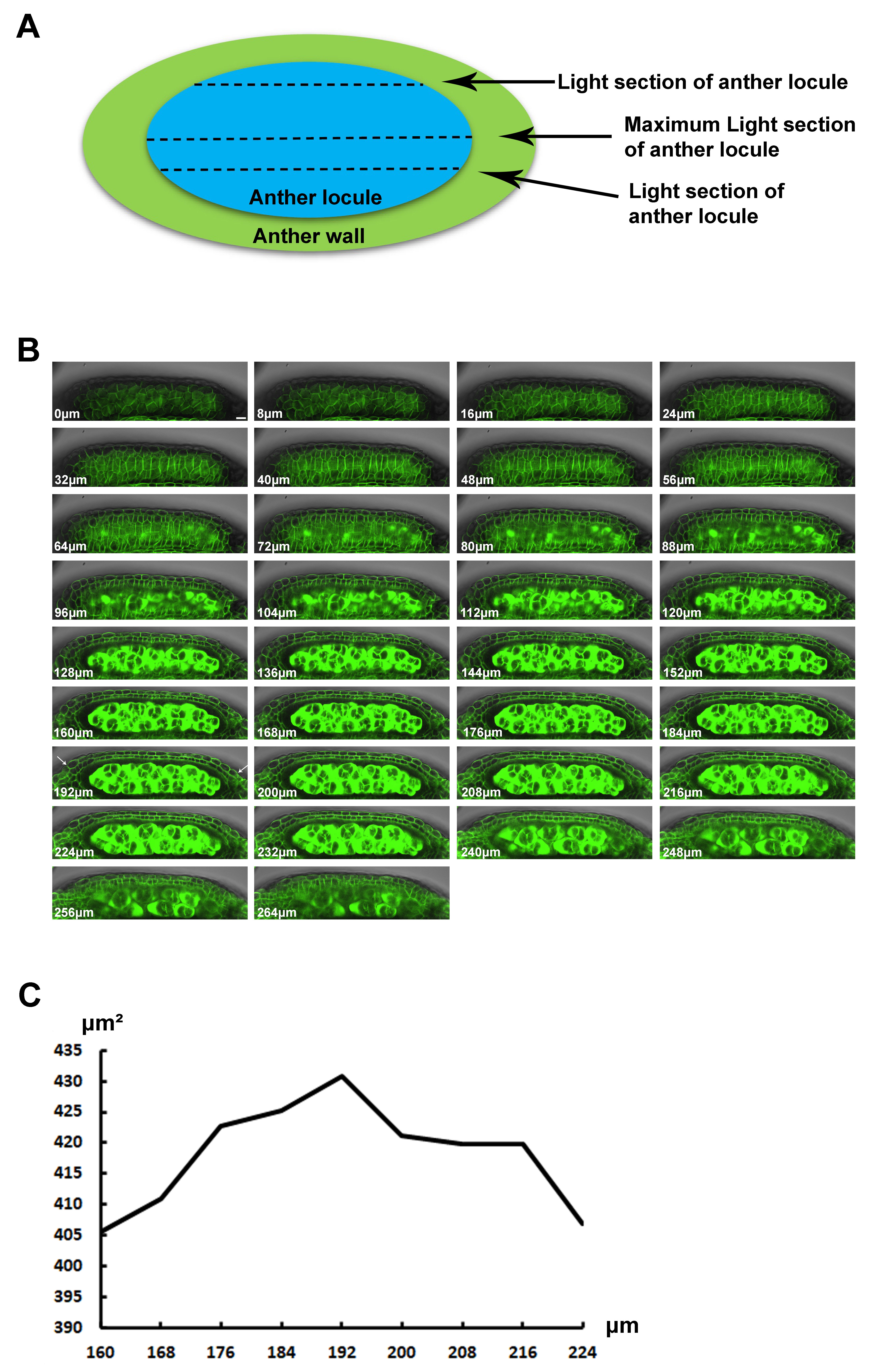

Supplement: Supplementary Figure 5 — The identification of the maximum light cross-section of intact anthers in stage 7. (A) The LSCM anther analysis model for cell division, cell area, and cell morphology. A Z-stack was performed to identify the maximum light sections of the intact anther. (B) The z-stack of a stage 7 anther. The epidermis layer was artificially set as 0 μm. The step size for the z-stack was 8 μm. Arrows show the region with four anther wall layers in the maximum light sections of the intact anther. Cell number counting was performed in this region. Five cells in the middle of the anther wall were used for the cell area and aspect ratio analysis. The CW signals were colored green. Scale bar = 10 μm. (C) Statistical analysis of the anther locule area in light sections for the identification of the maximum light section. The maximum light section was further used for other analyses. [file Image_5.jpg]
